# The Ketogenic Diet Reduces the Harmful Effects of Stress on Gut Mitochondrial Biogenesis in a Rat Model of Irritable Bowel Syndrome

**DOI:** 10.3390/ijms22073498

**Published:** 2021-03-28

**Authors:** Guglielmina Chimienti, Antonella Orlando, Angela Maria Serena Lezza, Benedetta D’Attoma, Maria Notarnicola, Isabella Gigante, Vito Pesce, Francesco Russo

**Affiliations:** 1 Department of Biosciences, Biotechnologies, and Biopharmaceutics, University of Bari Aldo Moro, Via Orabona 4, 70125 Bari, Italy; guglielminaalessandra.chimienti@uniba.it (G.C.); angelamariaserena.lezza@uniba.it (A.M.S.L.); vito.pesce@uniba.it (V.P.); 2 Laboratory of Nutritional Pathophysiology, National Institute of Gastroenterology “S. de Bellis” Research Hospital, 70013 Castellana Grotte, Italy; antonella.orlando@irccsdebellis.it (A.O.); benedetta.dattoma@irccsdebellis.it (B.D.); 3 Animal Facility, National Institute of Gastroenterology “S. de Bellis” Research Hospital, 70013 Castellana Grotte, Italy; maria.notarnicola@irccsdebellis.it; 4 Laboratory of Nutritional Biochemistry, National Institute of Gastroenterology “S. de Bellis” Research Hospital, 70013 Castellana Grotte, Italy; isabella.gigante87@gmail.com

**Keywords:** irritable bowel syndrome, ketogenic diet, mitochondria, animal model

## Abstract

Functional alterations in irritable bowel syndrome have been associated with defects in bioenergetics and the mitochondrial network. Effects of high fat, adequate-protein, low carbohydrate ketogenic diet (KD) involve oxidative stress, inflammation, mitochondrial function, and biogenesis. The aim was to evaluate the KD efficacy in reducing the effects of stress on gut mitochondria. Newborn Wistar rats were exposed to maternal deprivation to induce IBS in adulthood. Intestinal inflammation (COX-2 and TRL-4); cellular redox status (SOD 1, SOD 2, PrxIII, mtDNA oxidatively modified purines); mitochondrial biogenesis (PPAR-γ, PGC-1α, COX-4, mtDNA content); and autophagy (Beclin-1, LC3 II) were evaluated in the colon of exposed rats fed with KD (IBD-KD) or standard diet (IBS-Std), and in unexposed controls (Ctrl). IBS-Std rats showed dysfunctional mitochondrial biogenesis (PPAR-γ, PGC-1α, COX-4, and mtDNA contents lower than in Ctrl) associated with inflammation and increased oxidative stress (higher levels of COX-2 and TLR-4, SOD 1, SOD 2, PrxIII, and oxidatively modified purines than in Ctrl). Loss of autophagy efficacy appeared from reduced levels of Beclin-1 and LC3 II. Feeding of animals with KD elicited compensatory mechanisms able to reduce inflammation, oxidative stress, restore mitochondrial function, and baseline autophagy, possibly via the upregulation of the PPAR-γ/PGC-1α axis.

## 1. Introduction

Applying a feasible and straightforward study model to the pathophysiology of functional gastrointestinal disorders (FGIDs) is still challenging since FGIDs result from the complex interplay between several putative factors including genetics, biology, and psychological attitude. Thus, in recent years, to summarize and overcome this complexity, a “biopsychosocial model” has been conceptualized [1].

In this model, an important role is played by the enteric nervous system (ENS) that acts not only as a regulator of digestive and absorptive functions in the gut, but is also tightly connected with the central nervous system (CNS), constituting the brain–gut axis in which stress can modulate or activate several afferent and efferent connections [2].

Among FGIDs, irritable bowel syndrome (IBS) is a highly prevalent disorder affecting an estimated 10–15% of the worldwide population [3]. An inflammatory component has been suggested to contribute to the etiology of this syndrome [4], and miscommunication between the ENS and CNS has been evoked for the onset of several symptoms apart from the classical and major gastrointestinal (GI) ones (e.g., abdominal pain, diarrhea, and constipation). Based on this assumption, migraine, fibromyalgia, cycling vomiting syndrome, and mood disorders have all been considered functional comorbidities for IBS [5].

These functional GI and extra-GI alterations have recently been associated with low cellular energy metabolism, driven by defects in the bioenergetics supply and the intracellular mitochondrial network [6]. Emerging evidence supports a broader role of mitochondria in several GI disorders of both inflammatory and malignant nature [7,8,9]. Mitochondria have also been suggested to play a crucial role in the rescue from the stress-mediated symptoms, as these organelles are the source of the energy supply for adaptive responses [10].

Mitochondrial implications in IBS do not rely only on their role as a cell powerhouse, but also on the extensive crosstalk between these organelles and the intestinal microbiota [11,12], and recent studies have demonstrated that an altered balance of microbial populations in the gut lumen is associated with the IBS pathogenesis and gut–brain connection [13].

There is an increased interest in using low carbohydrate diets like the ketogenic diet (KD) in IBS treatment [14], even if the reports are still few and lack firm conclusions. The low carbohydrate content in KD has been demonstrated to be protective against the proinflammatory effects of saturated fatty acid intake, at least in the contest of a balanced medium-/long-chain triglyceride composition [15].

The high fat, adequate-protein, low carbohydrate KD reprograms metabolism, forcing the body to utilize fat as a source of energy as it occurs during the fasting state. Through ketogenesis, fatty acids are oxidized to ketone bodies in the liver mitochondria and then distributed via the blood to the other organs to be consumed as fuel. It is considered an effective treatment in neurological [16] and mood disorders [17], also given its ability to remodel the gut microbiota and, consequently, impact the brain–gut axis [16].

Pleiotropic effects of KD involve many targets of oxidative stress, inflammation, and mitochondrial function and biogenesis, so it is suggested as a dietary therapeutic approach in the mitochondrial Leber hereditary optic neuropathy [18] and for the impaired mitochondrial functions in the autism spectrum disorder (ASD) [19].

Limited research has explored the effects of chronic psychosocial stress [10] or early-life stress (ELS) on gut mitochondria [20], leading to the conclusion that mitochondrial activity was disturbed [10,20]. On these bases, the present study aimed to evaluate the KD efficacy in reducing the harmful effects of stress on gut mitochondrial functions.

To achieve this goal, newborn Wistar rats were exposed to ELS through maternal deprivation (MD) to induce IBS in adulthood. Different determinants for IBS were taken into account and studied in colon samples of rats exposed to ELS and afterward fed with KD or a standard diet (Std), along with unexposed control rats. In detail, the intestinal inflammation was evaluated by the levels of cyclooxygenase-2 (COX-2) and Toll-like receptor-4 (TRL-4). The antioxidant response and the cellular redox status were investigated by assaying the levels of cytoplasmic and mitochondrial superoxide dismutases (SOD 1 and SOD 2, respectively), the mitochondrial peroxiredoxin III (PrxIII), and the content of oxidatively modified purines in the D-loop region of mtDNA. Mitochondrial biogenesis was studied by assaying the protein levels of the ligand-inducible transcription factor peroxisome proliferator-activated receptors-γ (PPAR-γ) and its co-activator peroxisome proliferator-activated receptor-gamma co-activator-1 alpha (PGC-1α), along with markers of mitochondrial mass such as levels of cytochrome c oxidase, subunit4 (COX-4), and mtDNA relative content. Finally, as markers of autophagy, the levels of Beclin-1 and LC3 II were analyzed. 

## 2. Results

### 2.1. Evaluation of Markers of Inflammation and Determination of the Redox Status

**Figure 1:**
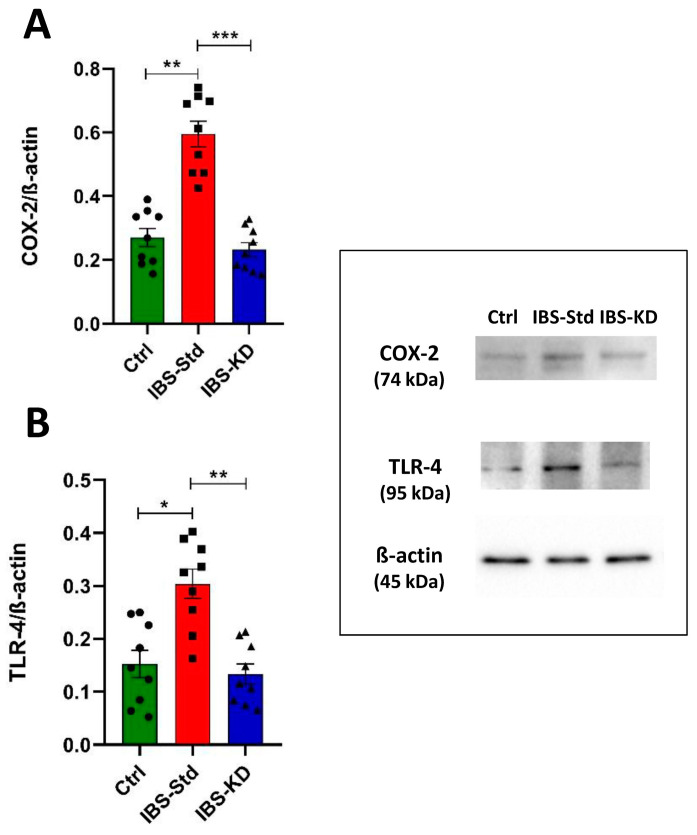
Western blot analysis of COX-2 (Panel (**A**)) and TLR-4 (Panel (**B**)) levels in colon samples of the control, IBS-Std, and IBS-KD rats, with each group consisting of four rats. Data were analyzed by Kruskal–Wallis analysis of variance and Dunn’s Multiple Comparison Test (* *p* < 0.05, ** *p* < 0.01, *** *p* < 0.001).

The etiopathogenetic inflammatory component in the IBS was evaluated by western blot analysis by determining the levels of COX-2 and TLR-4 proteins in the colon tissue of control, IBS-Std, and IBS-KD rats. As shown in Figure 1, the levels of COX-2 (Panel A) and TLR-4 (Panel B) were significantly about 2-fold higher in IBS-Std than the controls. Concerning both proteins, rats treated with KD showed values not significantly different from those in the controls.

The possible imbalance in redox status was determined by analyzing the levels of the cellular (SOD 1) and mitochondrial (SOD 2 and PrxIII) antioxidant enzymes, along with the content of oxidatively modified purines in mtDNA. 

**Figure 2:**
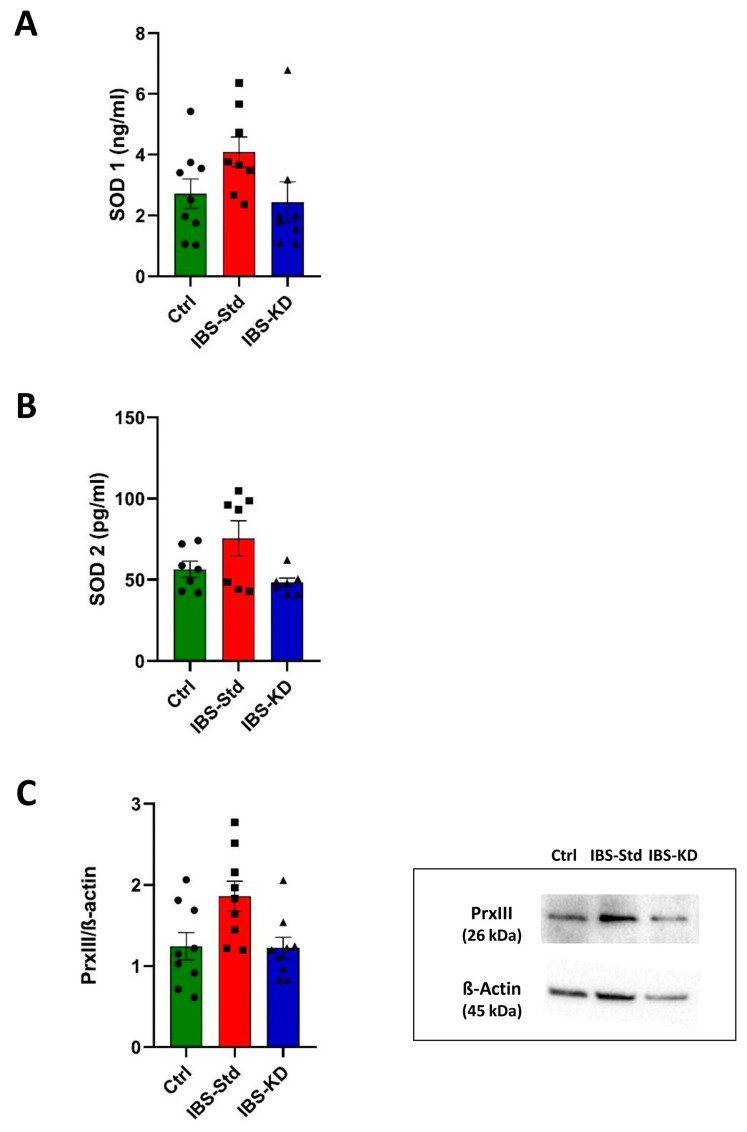
SOD 1 (Panel (**A**)) and SOD 2 (Panel (**B**)) levels, and western blot analysis of PrxIII (Panel (**C**)) in colon samples of the control, IBS-Std, and IBS-KD rats, with each group consisting of four rats. Data were analyzed by Kruskal–Wallis analysis of variance and Dunn’s Multiple Comparison Test.

As in Figure 2 (Panels A and B), the levels of SOD 1 and SOD 2, determined by the ELISA test, were higher, although not significantly, in IBS-Std rats and quite similar to controls in IBS-KD rats.

The levels of the mitochondrial ROS scavenger PrxIII, evaluated by western blot analysis, showed the same feature with a not significant increase in IBS rats fed with Std and values similar to those of controls in IBS rats fed with KD (Figure 2, Panel C).

**Figure 3:**
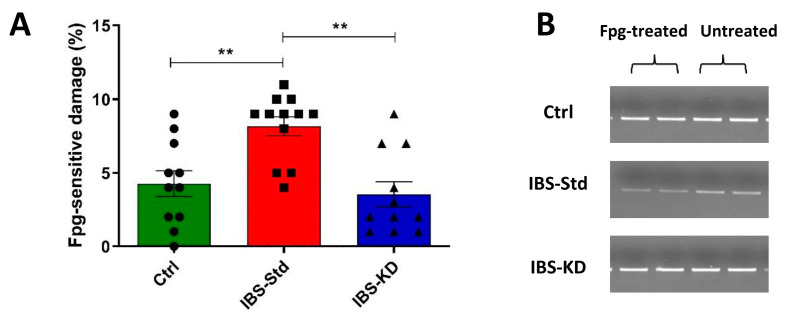
Incidence of oxidatively modified purines at the D-loop in the colon samples of the control, IBS-Std, and IBS-KD rats, with each group consisting of four rats. Data obtained using the oxidized purines-sensitive enzyme formamidopyrimidine DNA glycosylase (Fpg). Panel (**A**): the graph represents the ratio between Fpg-treated and untreated band intensities expressed as the complement to 100%. Data were analyzed by Kruskal–Wallis analysis of variance and Dunn’s Multiple Comparison Test (** *p* < 0.01). Panel (**B**): Representative gels showing amplicons obtained from Fpg-treated and untreated total DNA.

The incidence of oxidatively modified purines, mainly 8-oxo-deoxyguanosine (8-oxodG) at the D-loop, the major control region of mtDNA, was determined using the oxidized purines-sensitive enzyme formamidopyrimidine DNA glycosylase (Fpg). The incidence appeared to be approximately two-fold higher in IBS-Std rats than Ctrl rats, and the KD appeared to be able to reduce this content to values similar to those in the controls (Figure 3).

### 2.2. Mitochondrial Biogenesis

Levels of PPAR-γ, PGC-1α, COX-4 (evaluated by western blot analysis), and the mtDNA content (evaluated by qPCR) were determined.

**Figure 4:**
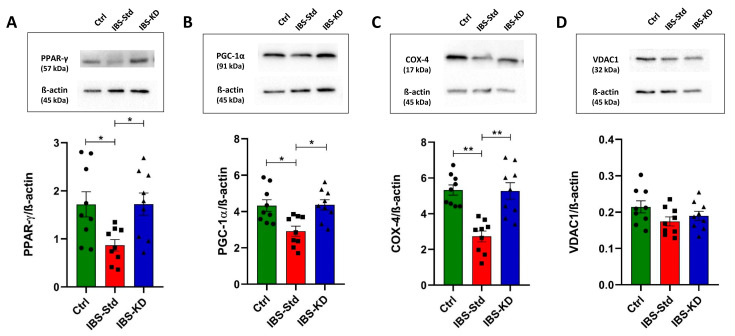
Western blot analysis of PPAR-γ (Panel (**A**)), PGC-1α (Panel (**B**)), COX-4 (Panel (**C**)), and VDAC1 (Panel (**D**)) levels in colon samples of the control, IBS-Std, and IBS-KD rats, with each group consisting of four rats. Data were analyzed by Kruskal–Wallis analysis of variance and Dunn’s Multiple Comparison Test (* *p* < 0.05, ** *p* < 0.01).

All the protein contents resulted in being significantly decreased by approximately two-fold in IBS-Std compared to the controls (Figure 4, Panels A–C).

The content of the specific loading marker VDAC1 was analyzed to exclude that the obtained findings could be derived from a different loading of mitochondrial proteins. No statistically significant differences were found among the three experimental groups (Figure 4, Panel D).

**Figure 5:**
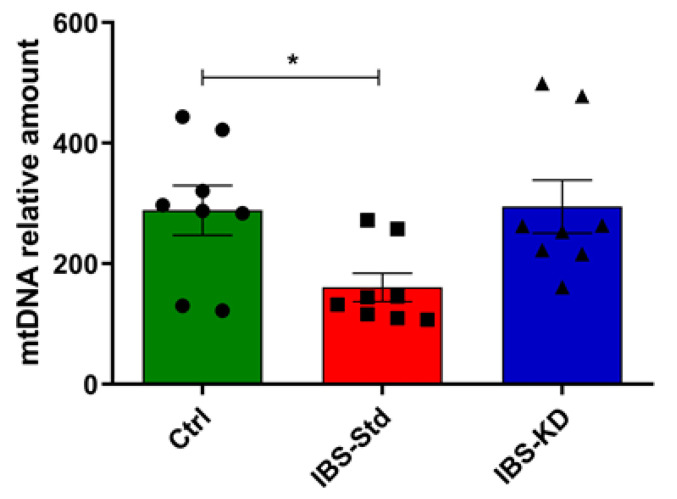
mtDNA content relative to the β-actin nuclear gene in colon samples of the control, IBS-Std, and IBS-KD rats, with each group consisting of four rats. Data were obtained using qPCR. Data were analyzed by Kruskal–Wallis analysis of variance and Dunn’s Multiple Comparison Test (* *p* < 0.05).

The mtDNA also appeared to be significantly reduced (about 2-fold) in IBS-Std than in the controls (Figure 5).

The KD was able to counteract such a reduction since the levels of all parameters were not statistically different from those in the control rats (Figure 4 and Figure 5). 

### 2.3. Evaluation of Markers of Autophagy

As a measure of autophagy, the protein content of Beclin-1 and LC3 II was determined by western blot analysis.

**Figure 6:**
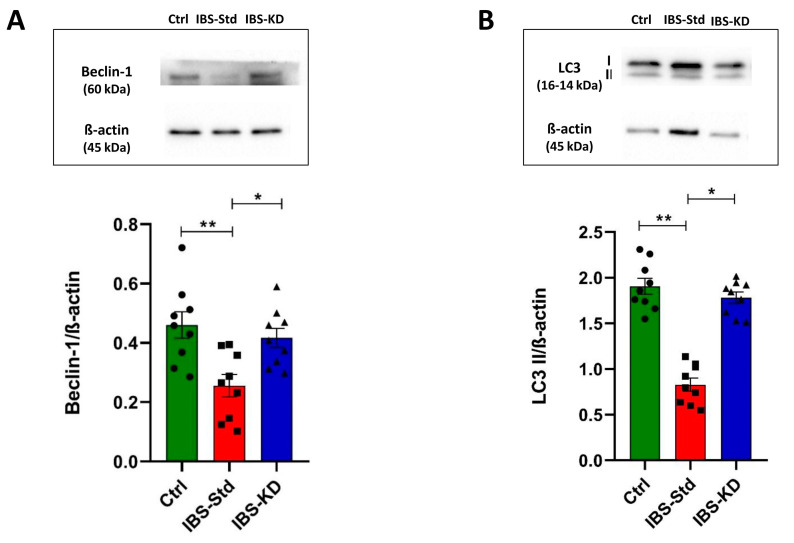
Western blot analysis of Beclin-1 (Panel (**A**)) and LC3 II (Panel (**B**)) levels in colon samples of the control, IBS-Std, and IBS-KD rats, with each group consisting of four rats. Data were analyzed by Kruskal–Wallis analysis of variance and Dunn’s Multiple Comparison Test (* *p* < 0.05, ** *p* < 0.01).

As shown in Figure 6, significant decreases (1.8 fold) in Beclin-1 (Panel A) and LC3 II (−60%) (Panel B) levels were observed in IBS-Std rats compared with the control ones. The ability of the KD to rescue from these variations and restore values as in the controls was evident.

## 3. Discussion

A broad range of intestinal pathologies involves mitochondrial dysfunction [7,8,9,10]. This study aimed to evaluate the KD efficacy in reducing the harmful effects of stress on gut mitochondrial biogenesis using an animal model mimicking the brain–gut axis alterations. The obtained findings support the possible involvement of gut mitochondrial abnormalities in the functional comorbidities associated with IBS [10,20].

One of the more significant results to emerge from this study is that rats exposed to ELS induced by MD and fed with a standard diet had an activated inflammatory response in the colon in adulthood, as shown by the higher levels of COX-2 and TLR-4 than those in the control rats. An abnormally higher expression of COX-2 in the colon has been associated with a worse prognosis in colorectal cancer patients [21], and this protein has been chosen as a pharmacological target to alleviate inflammation of post-inflammatory IBS [22].

Our group has already suggested TLR-4, a leading player in the activation of host immunity being a membrane receptor for LPS, as a useful circulating biomarker for the characterization of IBS subtype with prevalent diarrhea [23]. TLR-4 is also involved in the crosstalk between inflammation and autophagy given its implications in the molecular pathway leading to impaired autophagy, enhanced production of proinflammatory molecules, and oxidative stress in the gut [24].

As recently published elsewhere by our group [25], the histological analysis was performed in the same experimental animals, demonstrating the presence of a normal intestinal mucosa with intact epithelium in the control animals. 

In contrast, the IBS-Std animals showed a high inflammatory cell infiltration into both mucosa and submucosa, whereas the IBS-KD animals had mild infiltration of mixed inflammatory cells.

The oxidative hypothesis has been proposed as one of the pathological mechanisms involved in IBS [26]. However, the cytoplasmic antioxidant SOD 1 appeared not to be significantly activated in IBS rats fed with the standard diet. Since mitochondria are the primary cellular ROS source, these organelles are equipped with specific enzymes whose function is to activate an antioxidant response inside them to avoid an oxidative environment. This harmful condition could render the macromolecules therein more vulnerable to substantial damages. In the IBS-Std rats, the not significant increase of the mitochondrial specific scavengers of ROS (SOD 2 and PrxIII), along with the higher presence of oxidatively modified purines in the D-loop region of mtDNA, a hot spot for the damage [27], appeared to be indicative of the inability of these animals to set up an efficacious antioxidant response inside the mitochondria.

Indeed, mitochondria adopt several responses such as the activation of biogenesis, alteration of dynamics, and autophagy to adapt to fluctuating cellular conditions and maintain their proper functions [28]. The expression of nuclear-encoded mitochondrial genes is regulated by a network of transcription factors, co-activators, and co-repressors [29]. In this study, we evaluated the colonic content of the ligand-inducible transcription factor PPAR-γ and its non-enzymatic co-activator PGC-1α, a molecule at the intersection of myriad cellular pathways. 

PPAR-γ is primarily expressed in the colon and functions as an endogenous factor in several cellular functions like the metabolism and homeostasis of lipids and carbohydrates, the cell cycle regulation, and the cell differentiation, also being a regulator of bowel inflammation [30]. Decreased PPAR-γ expression has been described in intestinal inflammation [31], so thanks to its role in the interplay between inflammation and the immune system, the pharmacologic use of its agonists has been shown to improve IBS [32].

This transcription factor also regulates the gene coding for PGC-1α, and the PPAR-γ/PGC-1α axis upregulates the mitochondrial biogenesis to promote cellular functional recovery after injury [33,34]. The here shown evidence of lower levels of the transcription factor and its co-activator in IBS rats fed with the standard diet compared to the controls well fits with the observed COX-4 and mtDNA reduced levels. 

In line with data reported by other authors [10,20], our findings indicate dysfunctional mitochondrial biogenesis [35] in IBS-Std rats, which appeared to be associated with increased oxidative stress and inflammation in the gut. 

The downregulation of PGC-1α within the intestinal epithelium has already been demonstrated in human IBD and experimental murine colitis [36]. Cunningham et al. [36] reported that the reduced levels of PGC-1α were associated with failure in mitochondrial bioenergetics, leading to an impaired intestinal barrier and increased bacterial translocation that contribute to the pathogenesis of colitis. In their animal model, the authors also showed an early increase of PGC-1α, considered as an early adaptive response to oxidative stress that, however, finally failed due to ongoing insult.

Due to the crucial role of mitochondria as a cellular powerhouse, the quality of this organelle pool needs to be continuously controlled to ensure its proper functioning. The clearance of oxidatively modified macromolecules through the induction of autophagy/mitophagy represents an adaptive response when damaged mitochondria generate an excessive amount of ROS, which overwhelms the defenses. In this connection, a new function for the transcriptional co-activator PGC-1α in the adaptive stress response has emerged in the last years [37]. 

Beyond its function as the master regulator of mitochondrial biogenesis and activator of the antioxidant defense, this protein has a role as coordinator of the autophagic removal of damaged components [37]. The baseline autophagy is crucial for maintaining intestinal homeostasis, as demonstrated by the remarkably rapid mitochondrial turnover (0.7 days in the rat small intestine compared to 24.4 days in the rat brain) [38]. In recent years, autophagy has become a novel issue in GI pathologies [39], providing a further link between stress and the inflammatory status of the GI tract [40]. Actually, the role of autophagy in the inflamed intestinal epithelium is still under debate since available data are conflicting. In IBD patients, Wang et al. [40] showed enhanced intestinal autophagy that appeared to be associated with a worse course of the disease through the modulation of inflammation. More recently, Vincent et al. [41] proposed intestinal autophagy as a homeostatic mechanism to mitigate mitochondrial stress and dysfunction. In the present study, we evaluated the colonic levels of Beclin-1, a protein involved in the starting of autophagy, and the phosphatydilethanolamine-bound form (LC3 II) of the microtubule-associated protein 1 light chain 3 (LC3), which correlates to autophagosome levels. Due to the complexity of the autophagic process, two distinct autophagy markers involved in different steps of the process were evaluated in this study. The reduced levels of Beclin-1 and LC3 II we found in IBS-Std rats compared to the controls indicate the loss of autophagy efficacy in the former group of animals. Although caution is required in interpreting the results since total LC3 levels can change unpredictably [42], the overall agreement of the results obtained by the two markers allowed us to be confident in our interpretation. Considering the role of PGC-1α in the control of the maintenance of a functional mitochondrial pool through the processes of biogenesis, dynamics, and removal of damaged organelles [43], we can speculate that the reduced levels of this protein that we found are not sufficient to coordinate a proper adaptive response [33,34].

The effect of a ketogenic microenvironment, induced in an endogenous or exogenous manner like by the KD, is often studied to understand the pathogenesis of various intestinal inflammatory states. One of the effects resulting from the KD administration is the rise in the circulating ketone bodies. As a rule, the impact of KD consists of a shift toward proteolytic fermentation, leading to a reduction in intestinal mucosa inflammation [44].

Data from this study indicate the ability of KD to rescue from the stress-mediated mitochondrial dysfunction in the gut. In addition to allowing a higher ATP production [45], a complex network links nutritional ketosis and mitochondrial metabolism [46]. Several hypotheses have been proposed on the modes of actions of KD in improving mitochondrial functions. As for the neuroprotective role of KD, some authors have proposed that it could rely on the ability of ketone bodies to cross the blood–brain barrier [47] and activate the fatty acids and ROS sensitive uncoupling protein UCP2, able to reduce ROS production [48]. In liver samples, KD affects mitochondrial protein acetylation [49]. Moore et al. [50] demonstrated that KD, combined with exercise, can suppress de novo lipogenesis and increase mitochondrial biogenesis/content.

In contrast, Newell et al. [19] showed that in the liver of an animal model of ASD, KD increased mitochondrial turnover, thereby decreasing mtDNA. An increase in mitochondrial mass, without a change in oxidative status, has been reported in skeletal muscle tissue [51], whereas in mice defective for the mitochondrial pyruvate carrier, KD improved heart failure [52]. Data concerning the effects of KD in the colon mainly relate to its ability to reshape the architecture of bacterial communities in the gut, whose positive or negative effect is still to be definitively clarified [53].

An interesting result from this study appears to be the rescue of the levels of PPAR-γ in IBS rats fed with KD compared to IBS rats fed with the standard diet. To suggest a possible scheme of the action of KD on gut mitochondria, we can refer to the link existing between PPARs, nutrition, metabolism, and inflammation [54]. It has been reported that a high-fat diet can upregulate PPAR-γ [55] and KD increases fatty acids [56], which are endogenous ligands of this transcription factor [57]. When activated, PPAR-γ exerts its anti-inflammatory and antioxidant roles [30], which in this study was apparent from the values of markers of inflammation and oxidative unbalance that came back to levels similar to those of controls. Furthermore, in this more physiological intracellular environment, mitochondrial functions and baseline autophagy also appear to be restored, possibly via the PPAR-γ/PGC-1α axis, which is able to regulate biogenesis and clearance of damaged cellular components [33,34].

Here, we provide preliminary evidence of the KD efficacy in improving mitochondrial functions in the colon and thus reducing the harmful effects of stress in an animal model of IBS. These data need to be validated by further studies and surely replicated in human clinical trials.

## 4. Materials and Methods

### 4.1. Animals and Experimental Design

The study was approved by the Italian Ministry of Health (approval date: 28 November 2018, no. 901/2018-PR) according to European Union guidelines (Directive 2010/63/EU for animal experiments). 

The animals were housed at the animal facility of the National Institute of Gastroenterology “S. De Bellis” Research Hospital, Castellana Grotte, Bari, Italy. All the applied procedures followed the International Guidelines for the use of laboratory animals, minimizing animal suffering.

The animal model chosen is the newborn Wistar Rat subjected to ELS through MD to induce IBS in adulthood [58]. Postnatal Day 0 (PND 0) was considered as the birthday. Within PNDs 2 to 14, the puppies experienced MD for 3 h a day.

The experimental design provided that, after weaning, the animals subjected to MD were further divided into two subgroups, one group fed a standard diet (IBS-Std), and one group fed a low carbohydrate, high fat ketogenic diet (IBS-KD). A control group of animals without MD and fed a standard diet was also included (Table 1). 

Diets were supplied as pellets (4RF21 standard diet and KD purchased by Mucedola Srl, Settimo Milanese, Italy) and administered for ten weeks after PND 14 (Table 2). 

Rats were checked every day, evaluating different parameters regarding the degree of suffering and stress-induced experimentally (namely, blepharospasm, hollow cheeks, abnormal position of the ears and the whiskers, appetite loss, and liquid stools). Each parameter was recorded, attributing a score from 0 (absent) to 2 (evident) to calculate the possible onset of pain and suffering.

All animals in the study did not show any of the above parameters, except for a slowdown in the growth of puppies with MD, which showed lower weights than the control group puppies.

After treatment, the animals were sacrificed by anesthetic overdose, and colon samples were immediately removed and stored at −80 °C until assayed.

### 4.2. Western Immunoblotting

Protein extracts were obtained from colon tissue samples of control, IBS-Std, and IBS-KD rats using standard procedure [7]. Aliquots of 50 µg of total protein extracts from each sample were loaded into 4–15% pre-cast polyacrylamide gels (Bio-Rad, Milan, Italy) for western blot analysis. Anti-PrxIII (Ab FRONTIER, Seoul, Korea), anti-PGC-1α (Ab NOVUS, Centennial, CO, USA), anti-COX-2, anti-TLR-4, anti-PPAR-γ, anti-COX-4, anti-Beclin-1, anti-LC3, anti-VDAC1 (Abcam, Cambridge, UK), and anti-β-actin (Cell Signaling, Danvers, MA, USA) were used as primary antibodies. The proteins were detected by chemiluminescence (ECL, Thermo Scientific, Rockford, IL, USA), and the densitometric analysis of each protein-related signal was obtained using the Molecular Imager Chemidoc^TM^ (Bio-Rad, Milan, Italy) and normalized against β-actin expression. 

### 4.3. SOD 1 and SOD 2 Levels

The [Cu-Zn] Superoxide dismutase (SOD 1) and the Mitochondrial Superoxide dismutase (SOD 2) levels in colon tissue samples from control and treated Wistar rats were evaluated using the Rat SOD 1 and Rat SOD 2 Enzyme-Linked Immunosorbent Assay (ELISA) Kits (Fine Test, Wuhan, China), respectively, following the manufacturer’s instructions.

### 4.4. Determination of mtDNA Content 

Quantitative real-time polymerase chain reaction (qPCR) was used to determine mtDNA content relative to β-actin nuclear gene, as reported in Chimienti et al. [27]. Briefly, 6 ng total DNA as template and the following primers: mtDNA for 5′GGTTCTTACTTCAGGGCCATCA3′ (nt 15,785-15,806), mtDNA Rev 5′TGATTAGACCCGTTACCATCGA3′ (nt 15,868-15,847) (numbering according to GenBank^TM^ accession number AY172581, Rattus norvegicus, complete mitochondrial genome); β-actin For 5′CCCAGCCATGTACGTAGCCA3′ (nt 2181-2200), β-actin Rev 5′CGTCTCCGGAGTCCATCAC3′ (nt 2266-2248) (GenBank^TM^ accession number V01217.1, Rattus norvegicus, β-actin gene) were used.

### 4.5. Modified Purines Analysis

Oxidized purines were detected using formamidopyrimidine DNA glycosylase (Fpg) (New England Biolabs, Beverly, MA, USA) digestion of total DNA, as in Chimienti et al. [27]. The PCR amplification of the D-loop mtDNA region was conducted using the following primers: D-loop for 5′TCTGGTCTTGTAAACCAAAAATGA3′ (nt 15,302-15,325), D-loop Rev 5′TGGAATTTTCTGAGGGTAGGC3′ (nt 16,302-16,282) (accession number AY172581, Rattus norvegicus, complete mitochondrial genome) and 7.5 ng of Fpg-treated or untreated total DNA. The Fpg-treated and untreated band intensities ratio was evaluated and expressed as the complement to 100 (%). 

### 4.6. Statistical Analysis

Due to the non-normal distribution of the data, nonparametric tests were performed. Data were analyzed by Kruskal–Wallis analysis of variance and Dunn’s Multiple Comparison Test. Differences were considered significant at *p* < 0.05. All data represent the results of at least two independent experiments and are expressed as mean ± SEM. A specific statistical package for the exact nonparametric inference package (StataCorp. 2005; Stata Statistical Software: Release 9, College Station, TX, USA) was used.

## Figures and Tables

**Table 1:** Experimental groups: no irritable bowel syndrome (IBS) rats fed a standard diet (Ctrl); IBS rats fed a standard diet (IBS-Std); IBS rats fed a low carbohydrate, high fat ketogenic diet (IBS-KD). Maternal deprivation (3 h/day from Postnatal Day (PND) 2 to 14). Treatment (for 10 weeks after PND 14).

Group	Rats (Number)	Maternal Deprivation (3 h/Day from PNDs 2 to 14)	Treatment (for 10 Weeks after PND 14)
Ctrl	12	No	Standard diet
IBS-Std	11	Yes	Standard diet
IBS-KD	17	Yes	Ketogenic diet

**Table 2:** Composition of standard diet (4RF21) and ketogenic diet.

Analytical Constituents	Standard Diet (4RF21)	Ketogenic Diet
Moisture	12%	0%
Crude protein	18.5%	16.0
Crude oils and fats	3.0%	67.0
Crude fibers	6.0%	6.0
Crude ash	7.0%	4.5
**Nutritional additives**		
Vitamin A	28,500 I.U.	25,770 I.U.
Vitamin D3	1260 I.U.	2860 I.U.
Fe	180 mg	60 mg
Mn	54 mg	87.5 mg
Zn	67.5 mg	48.5 mg
Cu	11.7 mg	8.5 mg
I	0.9 mg	0.3 mg
Se	-	0.16 mg

## Data Availability

The data that support the findings of this study are available from the corresponding author upon reasonable request.
